# Water/ethanol extract of *Cucumis sativus* L. fruit attenuates lipopolysaccharide-induced inflammatory response in endothelial cells

**DOI:** 10.1186/s12906-018-2254-1

**Published:** 2018-06-25

**Authors:** Chiara Bernardini, Augusta Zannoni, Martina Bertocchi, Irvin Tubon, Mercedes Fernandez, Monica Forni

**Affiliations:** Department of Veterinary Medical Sciences – DIMEVET, University of Bologna, Via Tolara di Sopra 50, Ozzano Emilia, 40064 Bologna, Italy

**Keywords:** Endothelium, *Cucumis sativus* L., Inflammation, Hemeoxygenase-1, Cytokines, Angiogenesis

## Abstract

**Background:**

It is widely accepted the key role of endothelium in the onset of many chronic and acute vascular and cardiovascular diseases.

In the last decade, traditional compounds utilized in “folk medicine” were considered with increasing interest to discover new bioactive molecules potentially effective in a wide range of diseases including cardiovascular ones. Since ancient times different parts of the *Cucumis sativus* L. plant were utilized in Ayurvedic medicine, among these, fruits were traditionally used to alleviate skin problem such as sunburn irritation and inflammation. The main purpose of the present research was, in a well-defined in vitro model of endothelial cells, to investigate whether a water/ethanol extract of *Cucumis sativus* L. (CSE) fruit can attenuate the damaging effect of pro-inflammatory lipopolysaccharide (LPS).

**Methods:**

Cell viability, gene expression of endothelial cell markers, cytokines secretion and in vitro angiogenesis assay were performed on porcine Aortic Endothelial Cells exposed to increasing doses (0.02; 02; 2 mg/ml) of CSE in the presence of pro-inflammatory lipopolysaccharide (LPS 10 μg/ml).

**Results:**

CSE reduced LPS-induced cytotoxicity and decreased the cellular detachment, restoring the expression of tight junction ZO-1. The increase of TLR4 expression induced by LPS was counterbalanced by the presence of CSE, while the protective gene Hemeoxygenase (HO)-1 was increased. *Cucumis sativus* L. inhibited the early robust secretion of inflammatory IL-8 and GM-CSFs, furthermore inhibition of inflammatory IL-6 and IL-1α occurred late at 7 and 24 h respectively. On the contrary, the secretion of anti-inflammatory IL-10, together with IL-18 and IFN-γ was increased. Moreover, the in vitro angiogenesis induced by inflammatory LPS was prevented by the presence of *Cucunis sativus* L. extract, at any doses tested.

**Conclusions:**

Our results have clearly demonstrated that *Cucumis sativus* L. extract has attenuated lipopolysaccharide-induced inflammatory response in endothelial cells.

## Background

Vascular integrity contributes to the maintenance of the homeostasis of the whole organism [1]. The break of the vascular balance causes many pathological alterations, including cardiovascular diseases (CDVs), that represent the principle cause of death globally [2].

Among vascular cellular components, endothelial cells (EC) establish the inner lining of blood vessels and perform a pivotal role in the maintenance of the vascular integrity [1, 3–5]. Moreover endothelial cells have a key position in the beginning, progression, control and resolution of the vascular dysfunction [6–9]. Several endogenous and exogenous pro-inflammatory stimuli, such as lipopolysaccharide (LPS), induce “EC activation”. The phenotype of activated endothelial cells promotes phenomena of vasoconstriction, leukocyte adhesion, coagulation and thrombosis. This change involves the up-regulation of pro-inflammatory genes, including secretion of inflammatory cytokines and chemokines. If the pro-inflammatory status is not counterbalanced by the synthesis of protective molecules, the endothelial activation converts into the endothelial dysfunction and then in the vascular disease [10, 11].

In full accordance with the principle of “Replacement”, one of the commonly-accepted 3Rs rules (Replacement, Reduction and Refinement) for more ethical use of animals in experimental testing, primary culture of porcine Aortic Endothelial Cells (pAECs) were successfully used in many different in vitro models, preceding the in vivo, confirming swine as a relevant animal model for translational medicine [12–17].

In the last decade, traditional compounds utilized in “folk medicine” have been considered with increasing interest to discover new bioactive molecules potentially effective in a wide range of diseases including cardiovascular ones. Nevertheless, to support the traditional medicine use of these compounds, scientific informations regarding the phytochemical or biological activity are needed. [18, 19].

Cucumber (*Cucumis sativus* L.) is a popular vegetable crop member of the Cucurbitaceae family commonly cultivated for its edible fruits. Since ancient times, different parts of the cucumber plant have been employed in Ayurvedic medicine, among these, fruits are traditionally used to alleviate skin problem such as sunburn’s irritation and inflammation [20, 21]. Recently in vitro evidences [22] suggested that a *Cucumis sativus* extract show strong anti-oxidant capacity and ability to stability the membrane of human red blood. Moreover, Patil [23] demonstrated that aqueous extracts of *Cucumis sativus* is efficacious on inflammatory model of ulcerative colitis in in vivo model of Wister rats.

Nowadays no studies have investigated the effect of *Cucumis sativus* L. on vascular endothelial cells. Therefore, to provide new scientific evidence to support traditional medicine use of *Cucumis sativus* L., the main purpose of the present research was to investigate whether a water/ethanol extract of *Cucumis sativus* L*.* fruit (CSE) can attenuate the deleterious effects of LPS in in vitro model of endothelial cells.

## Methods

### Chemicals and reagents

Human endothelial SFM medium, heat inactivated FBS (Fetal Bovine Serum), antibiotic-antimycotic and Dulbecco’s phosphate buffered saline (DPBS) were purchased from Gibco-Life technologies (Carlsbad CA, USA).

RNA isolation was performed with NucleoSpin RNA kit (Macherey-Nagel GmbH & Co. KG, Düren Germany), iScript cDNA synthesis kit and iTaq Universal SYBR Green Supermix were used for cDNA synthesis and qPCR analysis (Bio-Rad Laboratories Inc., Hercules, CA, USA). All plastic supports were purchased from Falcon, Beckton-Dickinson.

A water/ethanol extract of *Cucumis sativus* L. fruit (CSE), titrated for total iminosugar acids content by HPLC-MS (2 g/100g), was kindly provided by Naturalea (Naturalea SA, Lugano, CH - Cuvrex batch number CE1501).

### Cell culture

Porcine Aortic Endothelial Cells (pAECs) were isolated and maintained as previously described by Bernardini and colleagues [12]. Briefly thoracic aortic traits were collected in a local slaughterhouse from adult pigs. After collection, thoracic aortic traits were washed with DPBS, ligated at the ends, and transferred to the laboratory within 1 h on ice. After ligation of all arterial side branches, aortas were cannulated with modified syringe cones and silicone tubes to set up a closed system. The vessels were repeatedly flushed with DPBS and then filled with a collagenase solution and incubated for 20 min at 37 °C. The cellular sospension were then centrifuged at 800 x *g* for 10 min. The cellular pellet was resuspended in 1 mL human endothelial basal growth medium (Gibco-Invitrogen, Paisley, UK) supplemented with 5% fetal bovine serum (Gibco-Invitrogen) and 1% antibiotics-antimicotics (Gibco-Invitrogen). Cell number and viability (85–90%) were determined using a Burker chamber under a phase-contrast microscope after vital staining with trypan blue dye. Cells were maintained in a logarithmic growth phase by routine passages every 2–3 days at a 1:3 split ratio. To confirm their endothelial origin, cultured cells were checked by immunocitochemistry for endothelial cell markers: CD31 and Caderine. Then cells were expanded till 20th passages. All experiments were performed with cells from the third to the eighth passage. The first seeding after thawing was always performed in T-25 tissue culture flasks (3 × 10^5^ cells/flask) and successive experiments were conducted in 24-well plates (qPCR and western blot analysis), in 96-well assay plates (cytotoxicity) and 8-well slide chamber for in vitro angiogenesis assay. Cells were cultured in Human endothelial SFM medium, added with FBS (5%) and antimicrobial/antimycotic solution (1×) in a 5% CO_2_ atmosphere at 38.5 °C.

### Cytotoxicity

Since the non-toxicity of the extract is a fundamental pre-requisite, we first tested the cytotoxicity of the CSE in a concentration range of 0.0002–2 mg / ml. No toxicity was showed at any doses tested.

pAECs were seeded in a 96 wells plate (approximately 3 × 10^3^ cells/well) and exposed to increasing doses of *Cucumis sativus* L. (CSE) (0.02; 0.2; 2 mg/ml) in presence of lipopolysaccharide (LPS) (10 μg/ml) (*E. coli* 055:B5, Sigma-Aldrich Co, St Louis, MO, USA) for 24 h. Cytotoxicity was evaluated by trypan Blue exclusion dye using Countess® II FL Automated Cell Counter (Life Technologies).

### Quantitative real time PCR for ZO-1, TLR4, HO-1

pAECs were seeded in a 24 wells plate (approximately 4 × 10^4^ cells/well) and exposed to increasing doses (0.02; 0.2; 2 mg/ml) of CSE in presence of LPS (10 μg/ml) for 1, 7 and 24 h. At the end of experimental times, treated or control cells were collected and stored until gene expression analysis.

Total RNA was isolated using the NucleoSpin®RNA Kit, and high quality RNA, with A260/A280 ratio above 2.0 was used for cDNA synthesis. Total RNA (500 ng) was reverse-transcribed to cDNA using the iScript cDNA Synthesis Kit in a final volume of 20 μL. Swine primers were designed using Beacon Designer 2.07 (Premier Biosoft International, Palo Alto, CA, USA). Primer sequences, expected PCR product lengths and accession numbers in the NCBI database are shown in Table 1.

**Table 1:** Primer sequence used for quantitative Real Time PCR analysis

Genes	Forward (5′-3′)	Reverse (5′-3′)	Product size (bp)	Accession Number
HPRT	GGACAGGACTGAACGGCTTG	GTAATCCAGCAGGTCAGCAAAG	115	AF143818
HO-1	CGCTCCCGAATGAACAC	GCTCCTGCACCTCCTC	112	NM_001004027
TLR4	CAGATACAGAGGGTCATGCTTTC	GGGGATGTTGTCAGGGATTTG	215	NM_001113039.1
ZO-1	AGTGCCGCCTCCTGAGTTTG	CATCCTCATCTTCATCATCTTCTACAG	147	AJ318101

Quantitative real-time PCR was performed to evaluate gene expression profiles in CFX96 (Bio-Rad) thermal cycler using SYBR green detection system. A master mix of the following reaction components was prepared in nuclease free water to the final concentrations indicated: 0.2 μM forward primer, 0.2 μM reverse primer, 1X iTaq Universal SYBR Green Supermix. One μl of cDNA was added to 19 μl of the master mix. All samples were analyzed in duplicate. The qPCR protocol used was: 10 min at 95 °C, 40 cycles at 95 °C for 15 s and at 61 °C for 30 s, followed by a melting step from 55 °C to 95 °C (80 cycle of 0.5 °C increase/cycle).

The expression level of interest genes was calculated as fold of change using the 2^-ΔΔCT^ method [24].

### Western blot for TLR4 and HO-1

pAECs were seeded in a 24 wells plate (approximately 4 × 10^4^ cells/well) and exposed to increasing doses (0.02; 0.2; 2 mg/ml) of CSE for 24 h. At the end of experimental time, cells were harvested and lysed in SDS solution (Tris–HCl 50 mM pH 6.8; SDS 2%; glycerol 5%). Protein Assay Kit (TP0300, Sigma) was used to determine the protein content of cellular lysates. Aliquots containing 20 μg of proteins were separated on NuPage 4–12% bis-Tris Gel (Gibco-Life-Technologies) for 50 min at 200 V. The proteins were then electrophoretically transferred onto a nitrocellulose membrane by Turbo Blot System (Bio-Rad). The blots were washed in PBS and protein transfer was checked by staining the nitro-cellulose membranes with 0.2% Ponceau Red. Non-specific binding on nitrocellulose membranes was blocked with 5% milk powder in PBS-T20 (Phosphate Buffer Saline-0.1% Tween-20) for 1 h at room temperature. The membranes were then incubated over-night at 4 °C with a 1:500 dilution of anti-HO-1 rabbit polyclonal antibody (SPA 896 StressGen Biotecnologies Corp, Victoria BC, Canada) and 1:1000 anti TLR4 mouse monoclonal antibody (NB100–56566 Novus Biologicals, Littleton, CO, USA). After several washings with PBS-T20, the membranes were incubated with the secondary biotin-conjugate antibody and then with a 1:1000 dilution of an anti-biotin horseradish peroxidase (HRP)-linked antibody.

The western blots were developed using chemiluminescent substrate (Super Signal West Pico Chemiluminescent Substrate, Pierce Biotechnology, Inc., Rockford, IL, USA) according to the manufacturer’s instructions. Chemidoc instrument using Quantity One Software (Bio-Rad) acquired the intensity of the luminescent signal of the resultant bands.

In order to normalize the HO-1 and TLR4 data on the housekeeping protein, membranes were stripped (briefly: the membranes were washed 5 min in water, then 5 min in 0.2 M NaOH and then washed again in water) and re-probed for housekeeping α-tubulin (1:500 of anti α-tubulin MA1–19162, Thermo Fisher Scientific, Rockford, IL, USA).

The relative protein content (HO-1 or TLR4/α-tubulin) was expressed as arbitrary units (AUs).

### Multiparametric enzyme-linked immunosorbent assay (ELISA) for cytokines and chemokines

Concentration of 13 cytokines and chemokines (GM-CSF, IFN-γ, IL-1α, IL-1β, IL-1ra, IL-2, IL-4, IL-6, IL-8, IL-10, IL-12, IL-18, TNF-α) was measured by quantitative multiparametric ELISA (Enzyme-linked immunosorbent assay). (Porcine Cytokine/Chemokine Magnetic Bead Panel kit, Milliplex Map Kit, EMD Millipore Corporation, Billerica MA USA), following the manufacturer’s instructions. The Luminex xMAP bead-based multiplexed immunoassay technology and MAGPIX instrument provided with xPONENT 4.2 software were used.

### Capillary-like tube formation assay

The experiments were carried out using 8-well slide chamber (BD Falcon Bedford, MA USA) coated with undiluted Geltrex™ LDEV-Free Reduced Growth Factor Basement Membrane Matrix. Extracellular matrix coating was carried out for 3 h in a humidified incubator, at 38.5 °C, 5% CO_2_. pAECs (8 × 10^4^ cells/well) were exposed to increasing doses (0.02; 0.2; 2 mg/ml) of (CSE) in the presence of LPS (10 μg/ml) for 24 h.

At the end of experimental time, images were acquired using a digital camera installed on a Nikon epifluorescence microscope (Nikon, Yokohama, Japan) and analyzed by open software Image J 64.

### Statistical analysis

Each treatment was replicated three times or six times (cytotoxicity) in three independent experiments. The data were analysed by a one-way analysis of variance (ANOVA) followed by the Tukey post hoc comparison Test. Differences of at least *p* < 0.05 were considered significant. Statistical analysis was carried out by using R software (http://www.R-project.org) [25].

## Results

### CSE prevented LPS-induced cell death and ZO-1 reduction

**Fig. 1:**
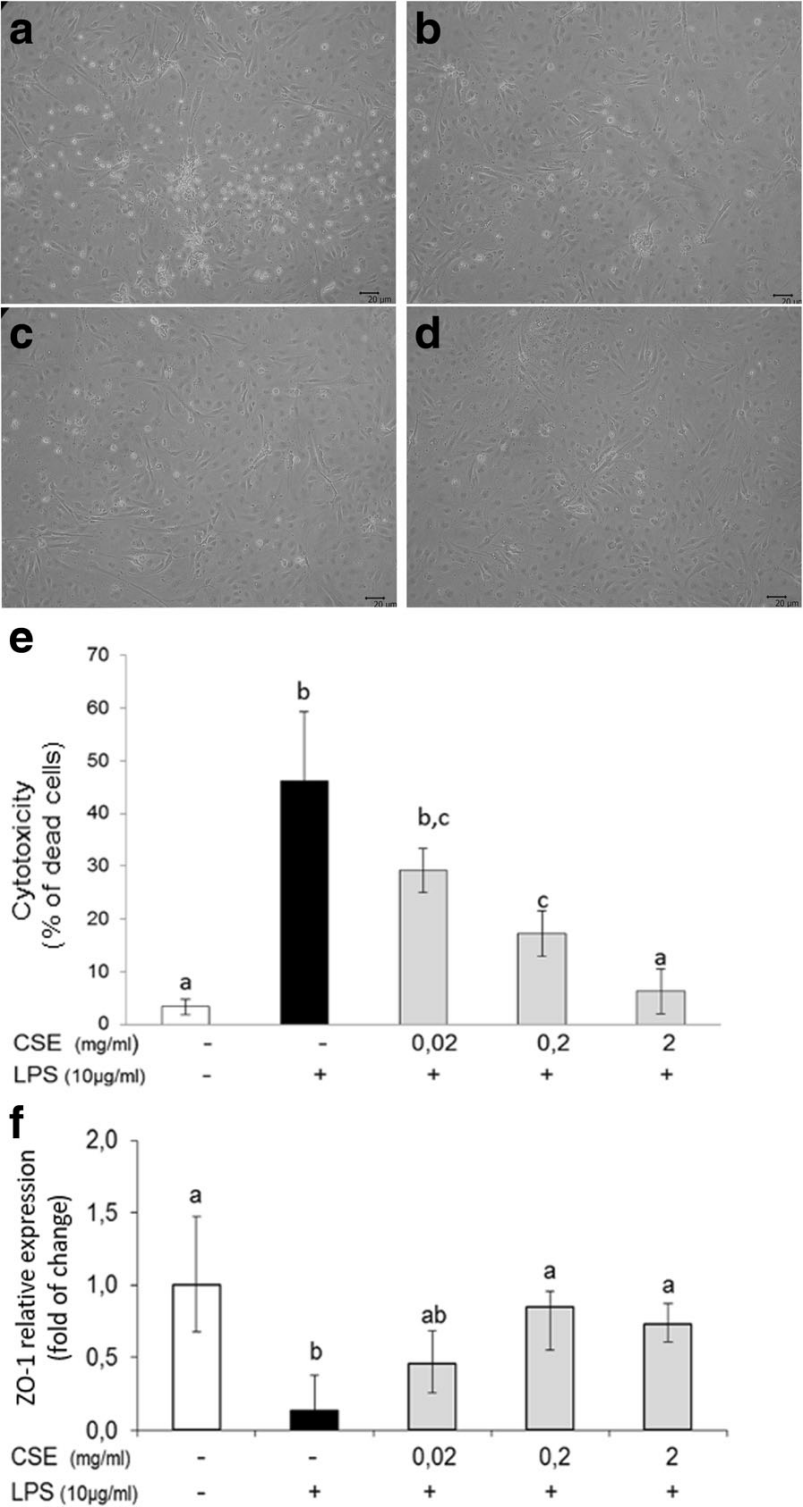
Effect of *Cucumis sativus* L. extract (CSE) on LPS-induced pAECs toxicity. **a** Representative images of pAECs morphology under LPS (10 μg/ml) stimulation (**b, c, d)** and in the presence of increasing dose of CSE (0.02; 0.2; 2 mg/ml). **e** Protective effect of CSE on LPS-induced cytotoxicity; data shown are representative of at least three independent experiments and represent the mean ± SEM. **f** CSE restored the LPS-induced decrease of ZO-1 mRNA expression; relative expression was calculated as fold of change in respect to the control cells and error bar represents the range of relative gene expression. Different letters above the bars indicate significant differences (*p* < 0.05 ANOVA post hoc Tukey’s test)

The protective effect of *Cucumis sativus* L. extract on LPS-induced toxicity was evaluated in pAECs. LPS treatment provoked an increased number of round and detached cells after 24 h (Fig. 1a), while CSE reduced the cellular detachment in a dose dependent manner (Fig. 1b-d). Cytotoxicity assay confirmed the ability of CSE to protect cells against LPS-induced cellular death (Fig. 1e). Moreover, we studied the expression of ZO-1, a critical component of tight junction scaffold; LPS induced the downregulation of ZO-1 gene expression, while CSE restored ZO-1 expression to control level at the intermediate and higher doses (Fig. 1f).

### Effect of CSE on TLR-4 and HO-1 expression

**Fig. 2:**
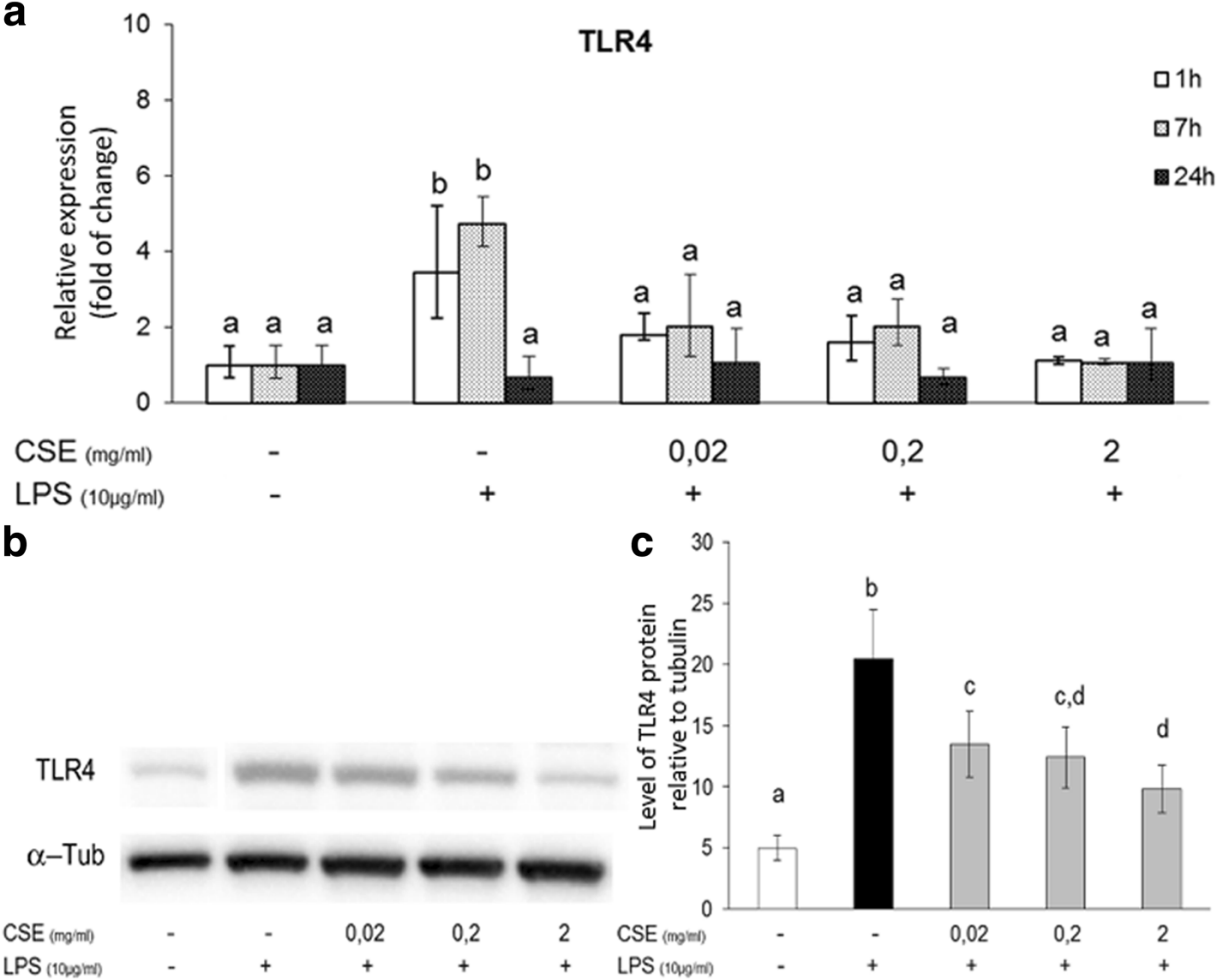
Effect of *Cucumis sativus* L. extract (CSE) on LPS induced TLR4 expression. **a** Expression of TLR4 mRNA in pAECs treated with LPS (10 μg/ml) for different time (1, 7, 24 h) in the presence or absence of increasing doses of CSE (0.02; 0.2; 2 mg/ml). mRNA expression of TLR4 is determined by quantitative PCR; relative expression was calculated as fold of change in respect to the control cells and error bar represents the range of relative expression. Different letters above the bars indicate significant differences. **b** Representative Western Blot of TLR4 and relative housekeeping α-tubulin were reported. **c** Expression of TLR4 protein in pAECs treated with LPS (10 μg/ml) in the presence or absence of increasing doses CSE (0.02; 0.2; 2 mg/ml); data shown are representative of at least three independent experiments and represent the mean ± SEM. Different letters above the bars indicate significant differences (*p* < 0.05 ANOVA post hoc Tukey’s test)

**Fig. 3:**
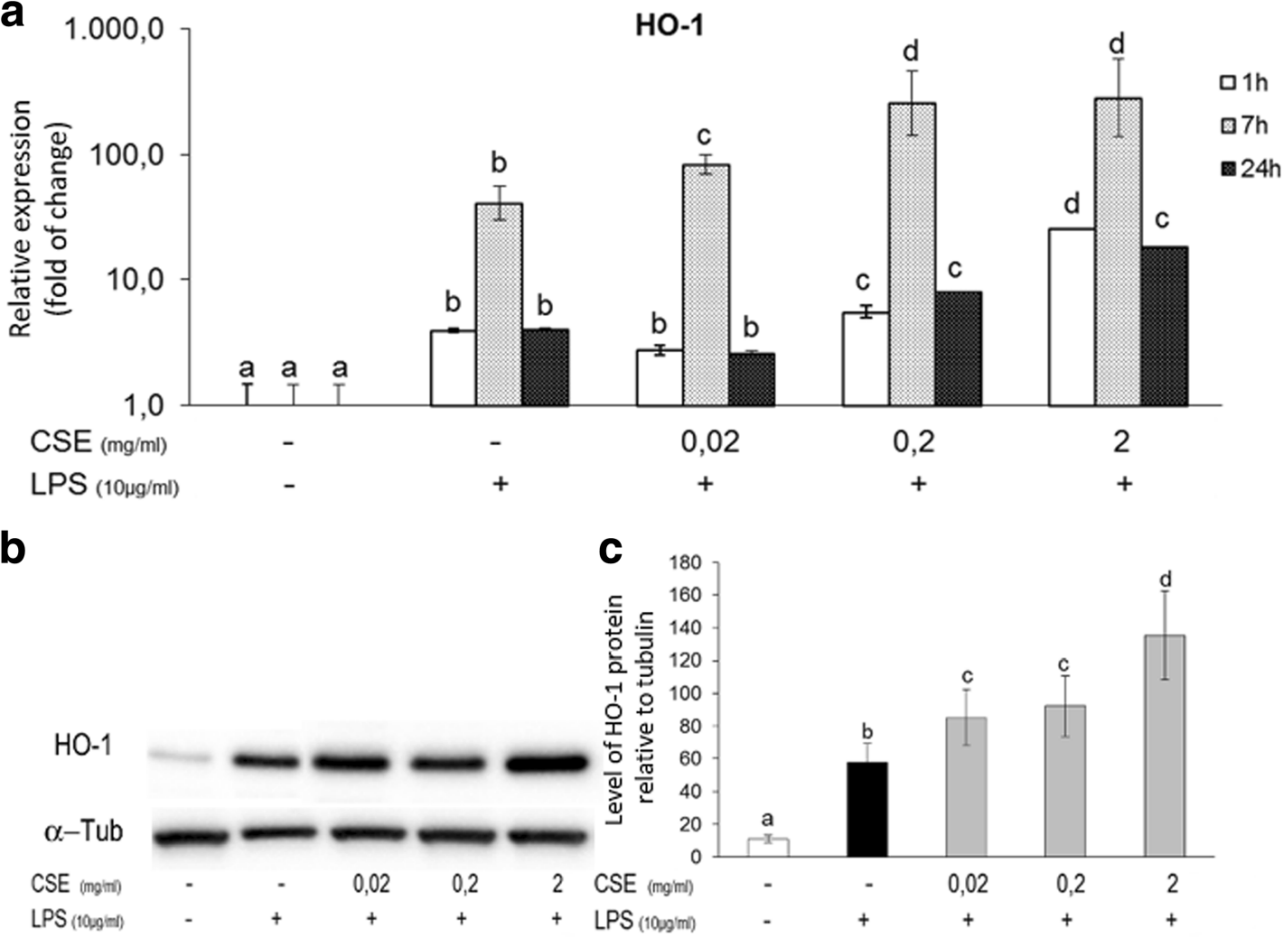
Effect of *Cucumis sativus* L. extract (CSE) on LPS induced HO-1 expression. **a** Expression of HO-1 mRNA in pAECs treated with LPS (10 μg/ml) in the presence or absence of increasing doses of *Cucumis sativus* L. extract (0.02, 0.2, 2 mg/ml). mRNA expression of HO-1 as determined by quantitative PCR. Relative expression was calculated as fold of change in respect to the control cells and error bar represents the range of relative gene expression. Different letters above the bars indicate significant differences. **b** Representative Western Blot of HO-1 and relative housekeeping α-tubulin were reported. **c** Expression of HO-1 protein in pAECs treated with LPS (10 μg/ml) in the presence or absence of increasing doses CSE (0.02; 0.2; 2 mg/ml); data shown are representative of at least three independent experiments and represent the mean ± SEM. (AU = Arbitrary Units) Different letters above the bars indicate significant differences (p < 0.05 ANOVA post hoc Tukey’s test)

We studied the effect of CSE on the expression of the Toll-like receptor 4 (TLR4) that is the main receptor for LPS recognition. LPS induced a significant increase of TLR4 mRNA after 1 and 7 h of treatment, while CSE inhibited its expression at all doses studied (Fig. 2a). This inhibitory effect was confirmed at protein level by western blot analysis as shown in Fig. 2b and c. Moreover, we studied the effect of CSE on the vascular protective molecule HO-1. LPS induced HO-1 expression in pAECs, additionally CSE increased HO-1 induction at both mRNA (Fig. 3a) and protein level (Fig. 3b and c).

### Effect of CSE on cytokine/chemokines secretion

To assess whether CSE could influence the LPS-induced secretion of inflammatory mediators we evaluated the presence of 13 cytokines/chemokines in the culture medium of pAECs treated with LPS in the presence or absence of CSE (2 mg/ml). LPS-treated endothelial cells released significant level of Il-6, Il-8, IL-10, IL-18, GM-CSF and IFN-γ. CSE significantly influenced these cytokines setting; in particular, the presence of the extract decreased the concentration of GM-CSF, IL-8 and IL-1α with a different kinetic, whereas the concentration of IL-10, IL-18 and IFN-γ was increased at each experimental point, as shown in Table 2. The level of IL-6 showed a precocious increase (1 h) in presence of CSE but after 7 and 24 h, cells treated with plant extract produced less IL-6 than cells only treated with LPS. IL-1β, IL-1ra, IL-2, IL-4, IL-12 and TNF-α were never detected in cell culture medium (data not shown).

**Table 2:** Levels of cytokines/chemokines in culture medium of pAECs stimulated with LPS (10 μg/ml) in the presence or absence of *Cucumis sativus* L. extract (CSE 2 mg/ml). Data shown are representative of at least three independent experiments and represent the mean ± SEM. Significant differences are indicated by (*p* < 0.05) *, and (*p* < 0.001) by ** *nd* not detectable

ng/ml	1 h		7 h		24 h	
	CSE-/LPS+	CSE+/LPS+	CSE-/LPS+	CSE+/LPS+	CSE-/LPS+	CSE+/LPS+
IL-1α	nd	nd	nd	nd	0.0592 ± 0.0019	0.0140 ± 00002**
IL-6	nd	0.1574 ± 0.0064	4.3163 ± 0.0893	3.3889 ± 0.0835*	8.0129 ± 0.3067	3.9270 ± 0.0335**
IL-8	6.3445 ± 0.4821	2.3809 ± 0.0434**	> 50	24.2300 ± 2.2100	31.5600 ± 3.8210*	25.1500 ± 2.7210
IL-10	0.0077 ± 0.0023	0.0355 ± 0.0010**	0.0187 ± 0.0025	0.0395 ± 0.0034*	0.0209 ± 00017	0.0400 ± 0.0056**
IL-18	0.0319 ± 0.0021	0.1796 ± 0.0049**	0.0572 ± 0.0011	0.1897 ± 0.0033**	0.0606 ± 0.0034	0.1913 ± 0.0056**
GM-CSF	0.1083 ± 0.0071	nd	0.1895 ± 0.0221	nd	0.2061 ± 0.0094	0.0021 ± 0.0037**
IFN-γ	0.0501 ± 0.0436	1.5529 ± 0.0292**	0.2654 ± 0.0889	0.5230 ± 0.0725*	0.0725 ± 0.0378	0.3522 ± 0.0340**

### Effect of CSE on LPS-induced angiogenesis

**Fig. 4:**
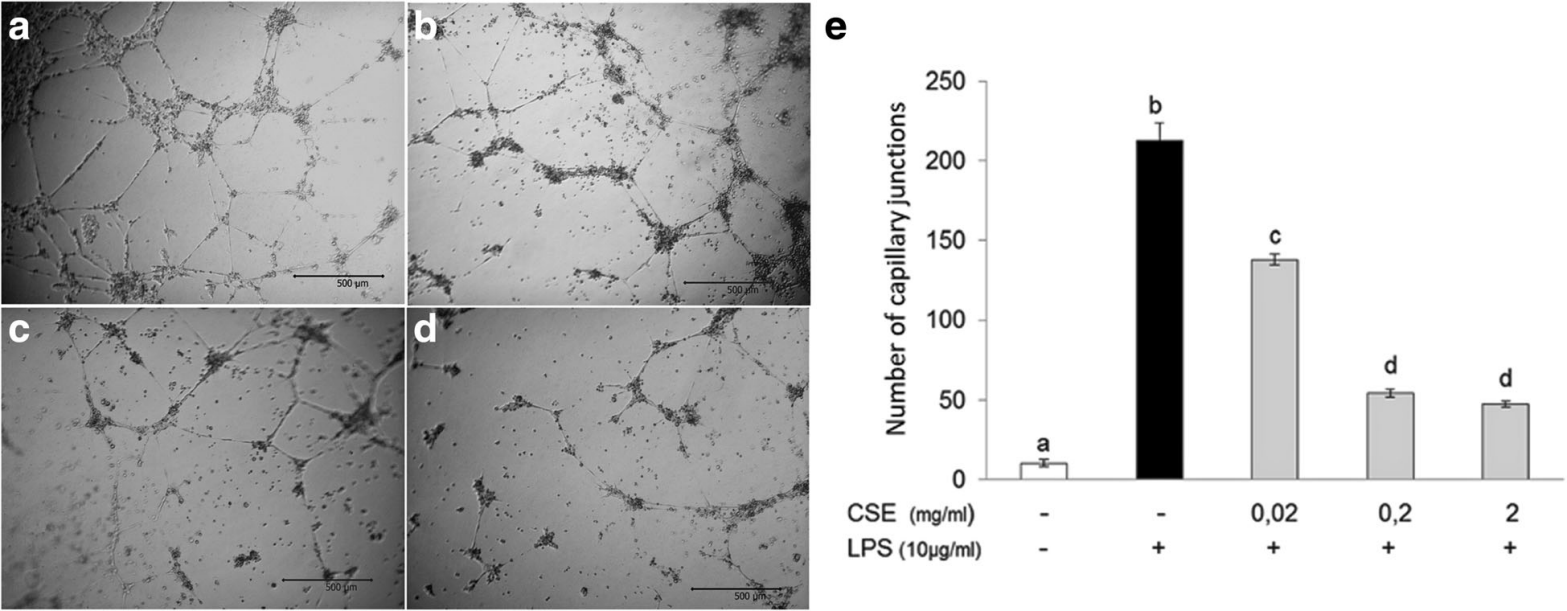
Effect of *Cucumis sativus* L. extract on LPS-induced angiogenesis. pAECs were cultured on a extracellular matrix with LPS (10 μg/ml) in the absence (**a**) or presence of increasing doses (0.02; 0.2; 2 mg/ml) of *Cucumis sativus* L. extract (**b, c, d** respectively) for 18 h. LPS induced a capillary like network (**a**, **e**) while CSE reduced LPS-induced angiogenesis at any doses tested (**b-e**). Data shown are representative of at least three independent experiments and represent the mean ± SEM. Different letters above the bars indicate significant differences (p < 0.05 ANOVA post hoc Tukey’s test)

We examined the effect of CSE on in vitro LPS-induced pAECs angiogenesis in an extracellular matrix-based assay. Cells cultured on extracellular matrix in the presence of pro-inflammatory LPS assembled in a complete tube and network formation (Fig. 4a and e), while the in vitro angiogenesis induced by inflammatory LPS was prevented by the presence of *Cucunis sativus* L. extract, at any doses tested (Fig. 4b-e).

## Discussion

It is widely accepted the key role of endothelium in the onset of many chronic and acute vascular and cardiovascular diseases. The shift from the healthy endothelium to the endothelial dysfunction is a complex process involving many different factors that starts with “the activation of endothelial cells”.

Recently, great effort is attempted to translate the potential activity of traditional compounds into the modern therapy, in a broad range of pathologies including cardiovascular disease [18]. *Cucumis sativus* L. is a very popular vegetable, native of India but nowadays commercially cultivated all over the world. Furthermore, since ancient time, Ayurvedic medicine has always used its fruits and seeds for their refrigerant, haemostatic tonic properties. It is now evident that fruits contain many interesting phyto-complex [20, 26] that makes it an interesting candidate for anti oxidant [27] and anti-inflammatory action [28] but the study of their effect still requires scientific supports.

The present study demonstrates that the protective effects reported for *Cucumis sativus* L., might be also mediated by its protective activity on the vascular endothelium.

Being the pig an excellent model for translational researches [29], in vitro approaches based on primary cell culture are required to better define the subsequent eventual in vivo activities to respect the 3Rs rules. We used in vitro cultures of porcine Aortic Endothelial Cells (pAECs), previously isolated and cultured by us to study vascular endothelial response to different shock, including LPS [12, 13, 30].

In the present research, LPS-induced effects on pAECs is contrasted by the contemporary administration of *Cucumis sativus* L. extract; in fact CSE protected endothelial cells against LPS-induced toxicity, in a dose dependent manner. Furthermore LPS reduced the expression of the tight junction molecule ZO-1, increasing the number of round and detached cells. Instead, *Cucumis sativus* L. extract, restored the ZO-1 expression, contributing to maintain the integrity of cellular tight junction, as confirmed by the reduction of cellular detachment.

It is well demonstrated that the exposure to LPS induces endothelial cell activation through the binding of a receptor complex that includes TLR4 [31–34]. In our model TLR4 expression is increased by LPS making cells more responsive to the stimulation, according to what seen by other researchers [35, 36]. In contrast, the presence of *Cucumis sativus* L. extract, inhibited the LPS-induced expression of TLR4.

The inflammatory signalling driven by TLR4 in endothelial cells goes through the activation of NF-κB and the consequently induction or shutdown of several genes including those for cytokines/chemokines synthesis [37].

The presence of CSE in the culture medium contrasted early the robust secretion of inflammatory IL-8 and GM-CSFs; while inhibition of inflammatory IL-6 and IL-1α occurred late at 7 and 24 h respectively. On the contrary, the anti-inflammatory IL-10 is increased together with IL-18 and IFN-γ.

Since the production and function of cytokines overlaps, what is the meaning of our results? Considering that in vivo endothelial cells mainly produce IL-6 and IL-8 and being, among the major functions of these cytokines, the induction of angiogenesis [11], the anti-angiogenic effect of CSE, evidenced by the in vitro-angiogenesis assay, is in agreement with the inhibition of these cytokines. Moreover interleukin 18 (IL-18), firstly described as a novel cytokine that stimulates interferon-γ (IFN-γ) production, possessed potent antitumor effects achieved by the inhibition of angiogenesis in vivo [38], so the increase of IL-18 in our model, could also contribute to a reduction in inflammatory angiogenesis.

Overall, our results demonstrate that the extract of *Cucumis sativus* L. influenced the secretion of cytokines/chemokines trough the reduction of TLR4 expression; moreover, the effect of this modulation inhibited the inflammation-induced angiogenesis. Overall, these important results suggest that *Cucumis sativus* L. extract could be a very interesting candidate in counteracting inflammatory pathologies in which TLR play a crucial modulatory role.

Furthermore, to avoid that the endothelial cell activation results in dysfunction, the induction of protective genes must be strictly regulated. Among protective genes, Hemeoxygenase (HO)-1, the rate-limiting enzyme in the heme catabolism, has been demonstrated to present important beneficial roles in the vasculature [39]; in particular HO-1 exerts antiapoptotic, antioxidants, antithrombotic and anti-atherogenic effects [39]. Our previous reports showed the LPS ability to induce HO-1 expression [12]; in the present research we demonstrated that *Cucumis sativus* L. extract increased the expression of vascular protective HO-1. Moreover, the role of HO-1 in angiogenesis is intriguing in fact HO-1 activity is necessary for VEGF-induced angiogenesis, whereas HO-1 has the opposite effect in the pathological angiogenesis [39]. Therefore, in our model, the increase of HO-1 could exert protective effect including the inhibition of LPS-induced inflammatory angiogenesis.

## Conclusions

Our results demonstrate the efficacy of a water/ethanol extract of *Cucumis sativus* L. fruit to protect vascular endothelial cells against LPS-challenge: decreasing LPS-induced TLR4 expression, influencing cytokines secretion, increasing the expression of protective HO-1. Moreover, the presence of *Cucumis sativus* L. extract inhibited the LPS-induced cellular toxicity and inflammation-induced angiogenesis. These impressive and robust results propose the *Cucumis sativus* L. extract as a promising natural compound in vascular endothelium protection.

## Abbreviations

CDVs: Cardiovascular diseases
CSE: *Cucumis sativus* L extract
EC: Endothelial cells
GM-CSF: Granulocyte-macrophage colony-stimulating factor
HO-1: Heme oxygenase 1
IFN-γ: Interferon γ
IL-10: Interleukin 10
IL-12: Interleukin 12
IL-18: Interleukin 18
IL-1ra: Interleukin-1 receptor antagonist
IL-1α: Interleukin 1α
IL-1β: Interleukin1β
IL-2: Interleukin 2
IL-4: Interleukin 4
IL-6: Interleukin 6
IL-8: Interleukin 8
LPS: Lipopolysaccharide
pAECs: Porcine aortic endothelail cells
TLR4: Toll-like receptor 4
TNF-α: Tumor necrosis factor α
ZO-1: Zona occludens-1
